# Identification of mRNA isoform switching in breast cancer

**DOI:** 10.1186/s12864-016-2521-9

**Published:** 2016-03-03

**Authors:** Wei Zhao, Katherine A. Hoadley, Joel S. Parker, Charles M. Perou

**Affiliations:** Department of Systems Biology, University of Texas MD Anderson Cancer Center, 77054 Houston, TX USA; Department of Genetics, University of North Carolina, 27599 Chapel Hill, NC USA; Curriculum in Bioinformatics and Computational Biology, University of North Carolina, 27599 Chapel Hill, NC USA; Lineberger Comprehensive Cancer Center, University of North Carolina, 125 Mason Farm Road, 27599 Chapel Hill, NC USA

## Abstract

**Background:**

Alternative splicing provides a major mechanism to generate protein diversity. Increasing evidence suggests a link of dysregulation of splicing associated with cancer. While previous genomic-based studies demonstrated the expression of a handful of tumor-specific isoforms, genome-wide alterations in the balance between isoforms and cancer subtypes is understudied.

**Result:**

We systematically analyzed the isoform-level expression patterns and isoform switching events of 819 breast tumor and normal samples assayed by mRNA-seq from TCGA project. On average, 2.2 isoforms per gene were detected and 67.5 % of detected genes (i.e. expressed) showed 1–2 isoforms only. While the majority of isoforms for a given gene were positively correlated with each other and the overall gene level, 470 pairs of isoforms displayed an inverse correlation suggesting a switching event. Most of the isoform switching events were associated with molecular subtypes, including a Basal-like-associated switching in CTNND1. 88 genes showed switching independent of subtypes, among which the isoform pattern of PRICKLE1 was associated with a large genomic signature of biological significance.

**Conclusion:**

Our results reveal that the majority of genes do not undergo complex mRNA splicing within breast cancers, and that there is a general concordance in isoform and gene expression levels in breast tumors. We identified hundreds of isoform switching events across breast tumors, most of which were associated with differences in tumor subtypes. As exemplified by the detailed analysis of CTNND1 and PRICKLE1, these isoform switching events potentially provide new insights into the post-transcriptional regulatory mechanisms of tumor subtypes and cancer biology.

**Electronic supplementary material:**

The online version of this article (doi:10.1186/s12864-016-2521-9) contains supplementary material, which is available to authorized users.

## Background

Gene expression patterns have been extensively studied due to the widespread use of DNA microarrays. Now with the advent of RNA-sequencing (RNA-seq), alternative splicing of genes can also be studied on a genome-wide level. Alternative splicing provides an additional layer for gene regulation and is a major mechanism to drive proteome diversity. Recent estimates indicate that the overwhelming majority of protein-coding genes in humans contain multiple exons, and more than 90 % of them produce multiple transcripts [1]. In normal tissues, alternative splicing is regulated according to the cell type, developmental stage, external stimulating signal, etc., and is coupled with nonsense-mediated mRNA decay pathway to regulate gene expression [2]. However, in several diseases including cancer, dysregulated alternative splicing can result in translation of aberrant proteins that can contribute to tumorigenesis. Although the definitive role of many mRNA isoforms is not clear, increasing evidence has suggested a link between alternative splicing and cancer causation [3, 4]. Investigations of alternative splicing patterns and their contribution to cancer will deepen our understanding of the oncogenic process, and potentially provide novel biomarkers [5].

A few cancer-related alternative splicing events have been extensively investigated. For instance, BCL-X gives rise to two functionally antagonistic isoforms: an anti-apoptotic isoform BCL-X_l_ and a short pro-apoptotic isoform BCL-X_s_. The up-regulation of BCL-X_l_ and/or down-regulation of BCL-X_s_ has been observed in several cancer types [6–8]. Another well-characterized gene, MDM2, expresses a remarkably complex splicing pattern. Isoforms that lack part of the p53-binding domain are unable to form p53-MDM2 interactions to regulate its degradation [9], leading to p53-dependent effects of gene expression in tumors [10–13].

In breast cancer, microarray and qRT-PCR-based studies have identified genes that express multiple splice variants including CD44, ESR1, ESR2, TP53, SYK, BRCA1 [14–16], and some of these are associated with specific breast cancer subtypes [16]. RNA-seq technology offers an accurate and unbiased approach to explore the heterogeneity of mRNA splicing on the global scale. Recent RNA-seq-based studies provided catalogues of alternative splice variants that are specific to tumor types, cell lines, or a subpopulation of primary tumors [17, 18], but most of the discovery was based on small cohorts. Currently, the increasing accumulation of RNA-seq data published by large consortiums, coupled with advanced statistical and computational tools, enables the extensive exploration of the diversity of alternative splicing with higher confidence. Here, we performed genome-wide analysis on a set of 819 breast tumors and normal tissues from the Cancer Genome Atlas (TCGA) [19]. We found that thousands of genes show alternative splicing, but that most isoforms of a gene are highly correlated in their overall gene expression patterns. Interestingly, only a small set of genes displayed dramatic isoform switching events.

## Results

### General isoform expression characteristics across 819 breast samples

In order to begin to study the complexity of mRNA isoform diversity, we used the isoform level expression data from 728 breast tumors and 91 normal breast tissues from TCGA breast data set (TCGA BRCA) [19, 20]. The summary of patient characteristics for this sample set is presented in Additional file 1: Table S1. Transcript abundance was estimated by RSEM [21], based on the UCSC known genes annotation (GAF2.1), which consists of 20,531 genes and 73,599 previously determined transcript definitions. In this paper, we used these known isoform definitions and did not perform any *de novo* isoform discovery or derive any new isoform definitions. In addition, we used RSEM to assign expression values to individual isoforms, which is a method where the common exon reads are proportionally assigned based upon the ratio of reads mapping to isoform unique regions.

**Fig. 1:**
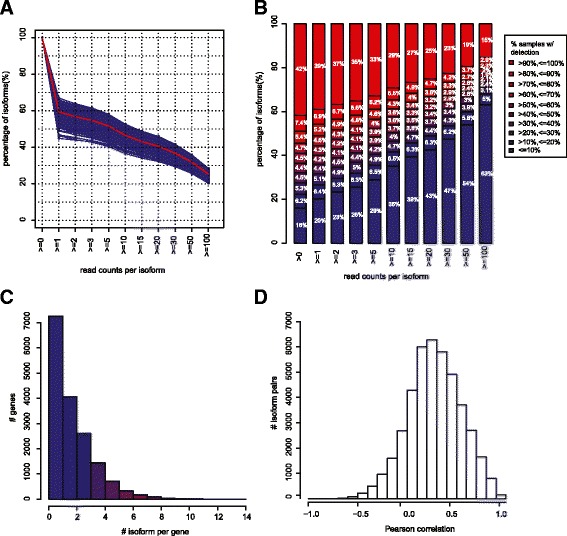
General isoform expression characteristics across 819 breast samples. **a** Proportion of detected isoforms using distinct cutoff to call isoform detection in 819 breast samples (blue lines) and on average (red line). **b** Distribution of isoforms expression across samples using different cutoff for isoform detection. **c** Distribution of the number of detected isoforms for each gene. **d** Pearson correlation of the abundance of detected isoform pairs of the same gene

Across the 819 samples, an average of 40.5 % of the 73,599 transcripts had less than one normalized read count and 25.5 % isoforms were detected in less than 10 % samples (Fig. 1a and b); these were not used for any subsequent analyses. 25.4 % of transcripts had more than 100 reads and approximately 35.1 % of transcripts were expressed in more than 90 % patients. To restrict the degree of sparsity while including the majority of the detected transcripts, analyses in this study were performed on the set of transcripts with at least 3 normalized read counts (i.e. detected) in more than 60 % patients, which included 37,267 isoforms from 16,765 genes (see Methods).

### Complexity and consistency of isoform expression

Of the 16,765 genes detected, 7258 expressed only 1 isoform, 4062 expressed 2 isoforms, and 5445 expressed 3 or more isoforms (Fig. 1c). According to this analysis, the majority of genes (67.5 %) showed simple isoform patterns of only one or two isoforms. In contrast, 43 genes were associated with more than 10 detected isoforms, which suggests that alternative splicing is potentially crucial in regulating the function of these genes (Additional file 2: Table S2). Functional analysis of these 43 genes using DAVID Tools [22] indicates a moderate enrichment of GO terms of cell cycle process, chromatin modification, apoptosis and DNA repair (Additional file 3: Table S3).

Despite the expression of isoforms for thousands of genes, the vast majority of alternatively spliced transcripts were correlated with the other transcripts of the same gene. The median Pearson correlation score of the 42,356 alternatively spliced isoform pairs was 0.28, with only 14.2 % pairs of isoforms negatively correlated (Fig. 1d). In fact, there were ~14,000 isoform pairs where the correlation was >0.4. There was not a significant association between the correlation of isoform pairs and the isoform expression level, or the isoform lengths (data not shown), although we do note that it is more likely to detect multiple isoforms in genes that show moderate to high expression levels.

**Fig. 2:**
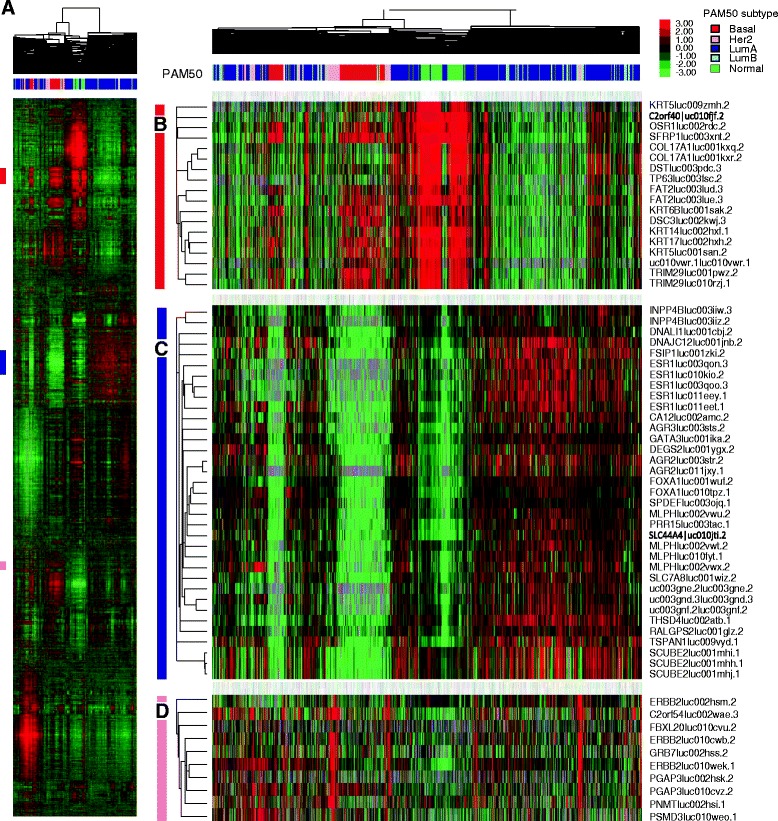
Hierarchical clustering analysis of the top 6000 most variably expressed transcripts. **a** Overview of the clustering diagram of 728 breast tumors and 91 normal samples. Gene signatures of (**b**) basal, (**c**) luminal and (**d**) Her2 subtype were identified, with multiple isoforms from the same biomarker genes co-clustered

On a global scale, the isoform expression patterns recapitulate the genomic profiles taken from the gene level data, which is not surprising given the overall high correlation in expression observed between isoforms of the same gene. To examine isoform level expression patterns, we performed unsupervised hierarchical clustering of the top 6000 most variably expressed transcripts (Fig. 2a). The intrinsic breast cancer subtypes, and their defining gene sets, could be clearly identified including basal-like, luminal and HER2-Enriched expression signatures (Fig. 2b-d); however, now these gene clusters were often populated with multiple isoforms from the same gene that were typically co-clustered together, such as multiple isoforms of ESR1 and SCUBE2 in the luminal cluster (Fig. 2c), and multiple isoforms of HER2/ERBB2 in the HER2 amplicon cluster (Fig. 2d). Overall, no new gene clusters/signatures were identified when using isoform level data, but instead, greater detail was included in all previously identified gene clusters.

### Identification of isoform switching pairs

The majority of alternatively spliced isoforms from the same gene showed correlated expression, therefore, we purposely searched for those genes that showed “isoform switching” or alternative usage of isoforms; we define “switching” as isoform pairs that were negatively correlated. Given the sparsity of much of the isoform data, we focused on a specific type of isoform switching, namely the switching of the predominant isoform to an alternative isoform(s), where the predominant isoform was identified as the transcript with the greatest upper quartile abundance for each gene. 2,110 pairs of predominant/alternative isoforms showed significant negative correlation based on a linear regression model.

We noted, however, that there was at least one technical effect that was dramatically affecting this apparent “switching” result. A difference in RNA integrity (i.e. RIN score) contributed to a large proportion of the 2,110 predominant/alternative switching events. Hierarchical clustering of the expression data using the 2,110 pairs of isoforms separated the samples into two clusters that were significantly associated with RIN even in this set of TCGA samples where all but two normal samples had RINs >7 (Additional file 1: Table S1, Additional file 4: Figure S1A and B). Two groups of isoforms showed significantly high and low expression in the cluster of samples with low RINs (aka, sample Cluster 1) respectively, which represent 917 pairs of isoform switching (Additional file 5: Table S4). Among them, 893 highly expressed isoforms were the shorter isoforms of the pairs. Therefore, it suggests that the observed relatively higher level of short isoforms is potentially caused by loss of RNA integrity. After adjusting for the RIN bias, a modified linear regression model identified 470 pairs of isoform switching candidates from 452 genes (Additional file 4: Figure S1C, Additional file 6: Table S5). Functional ontology analysis of these genes suggests the involvement of a multitude of biological pathways including RNA processing (i.e. splicing), protein localization, cell cycle, methylation and chromatin modification (Additional file 7: Table S6); thus as would be expected, our analysis identified many genes previously known to undergo RNA processing/splicing.

### Investigation of isoform switching: subtype specific events

Previous efforts to catalog alternative splicing events by comprehensive microarray-based screening have revealed that many such events are specific to tumor subtypes [23]. Therefore, we sought to determine whether the 470 switching candidates were ubiquitous in all breast samples or were unique to certain subtypes. Every molecular breast cancer intrinsic subtype [24] was correlated with a number of switching events, with the basal-like subtype exhibiting the most compared to the others (Table 1, Additional file 6: Table S5). 229 pairs of isoforms from 224 genes displayed isoform switching in basal-like samples as compared to luminal (Additional file 8: Table S7). Intriguingly, among them, 49 pairs did not show a difference at the gene expression level (Additional file 8: Table S7), which highlights that for a small number of genes, alternative splicing provides additional genetic complexity that is not reflected by total gene expression levels.

**Table 1:** Subtype specificity of isoform switching pairs

	subtype-associated isoform pairs	subtype-associated genes	subtype-associated isoform pairs, no difference in gene abundance
Basal	246	363	55
Her2	105	235	51
LumA	196	308	56
LumB	127	263	49
Normal	239	390	36
Luminal vs. Basal	229	361	49

**Fig. 3:**
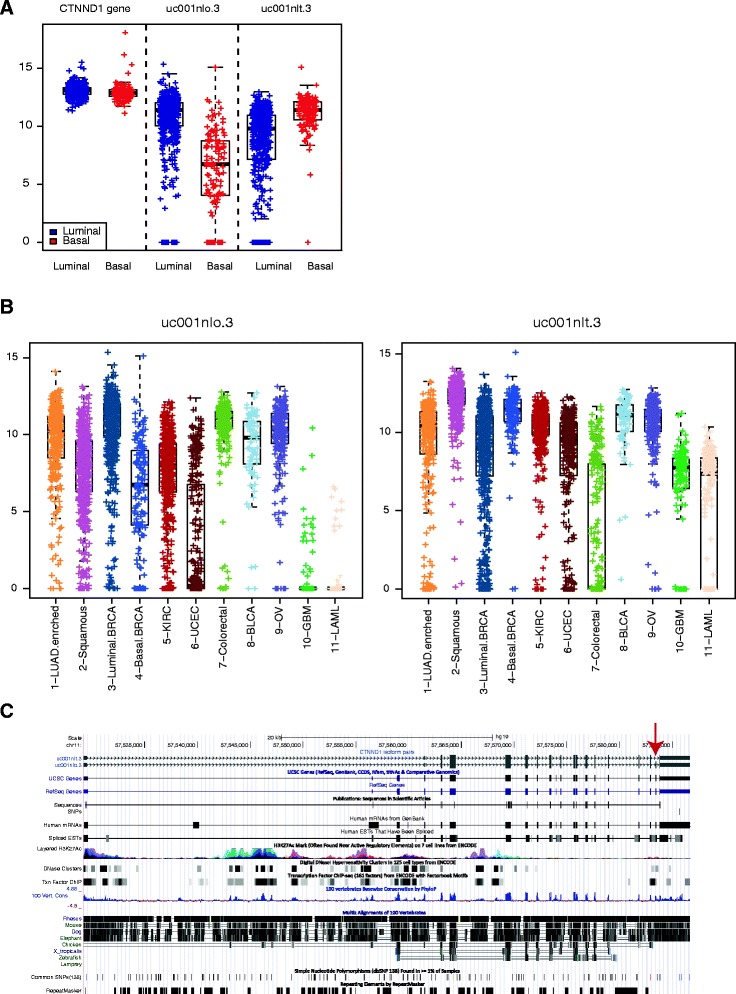
Isoform expression and gene structure of CTNND1. **a** Gene and isoform expression of CTNND1 in the TCGA BRCA dataset. The total gene expression of CTNND1 provides comparable level in luminal and basal tumors, whereas the abundance of two CTNND1 isoforms, uc001nlo.3 and uc001nlt.3, exhibit inversely correlated differential expression in luminal and basal tumors. **b** Isoform expression of CTNND1 in the TCGA PanCan12 data set. The expression pattern of uc001nlo.3 and uc001nlt.3 pairs is similarly observed in the PanCan12 Luminal BRCA and Basal BRCA tumors, as well LUAD and Squamous clusters. **c** CTNND1 gene structure. The two isoforms are transcribed from the same transcription start site and provide alternative splicing in exon 20 (red arrow)

Recently Hoadley et al. [25] demonstrated that the breast basal-like subtype showed striking similarity with squamous cancers on many genomic levels including gene expression patterns, somatic mutations and DNA copy number alterations. This commonality even applied to some alternatively spliced genes/transcripts including the well-known ΔNp63. The TCGA PanCan12 Squamous Genomic Group contained Head and Neck Squamous (HNSC), Lung Squamous (LUSC), and a few Bladder cancers with squamous features (BLCA). We therefore sought to examine whether these breast basal-like tumor associated isoform switching events were similarly observed in the TCGA Squamous Genomic Group tumors. We analyzed all 3308 tumor samples of the TCGA PanCancer12 study [25] for these basal-like isoform patterns. 40/229 basal-like associated isoform pairs showed consistent switching patterns between Squamous Genomic Group and PanCan Lung adenocarcinoma (LUAD)-enriched Group, and between PanCan Luminal Breast versus Basal-like Breast Groups, similar to what was observed here when comparing the basal-like and luminal breast subtypes (Additional file 8: Table S7). For example, CTNND1 had significant differences in comparing the expression pattern of a pair of isoforms, both with high detection rate, in luminal and basal-like samples, even though no statistical difference was seen in the total gene expression level comparison (Fig. 3a); this same switching pattern was also seen in the PanCan12 3308 tumor data set when we focused on the Squamous Genomic Group versus LUAD-enriched Group (Fig. 3b), thus suggesting that this pattern is conserved across related cell and/or tumor subtypes. CTNND1 may generate up to 30 isoforms by alternative inclusion/exclusion of 21 exons. Among them, 14 isoforms were detected in breast tumors for this study. Despite this complex splicing pattern, only the isoform pair of uc001nlo.3 and uc001nlt.3 was identified to have the switching event. These two transcripts both use the same ATG start site, but differ in the alternative inclusion of exon 20 of the gene, where a nuclear export sequence (NES) is located (Fig. 3c).

To exclude the possibility that the observation was caused by ambiguous read alignment, we validated the splicing pattern by *de novo* assembly (Additional file 9: Figure S2A). 10 samples that provided the highest relative expression level of either isoform respectively were pooled and *de novo* assembled to dissect the gene structure. In both subsets, only contigs consistent with the ‘sample-specific predominant isoform’ (i.e. the isoform with relatively high expression level in the 10 tumor samples being tested) were identified. This result offers additional evidence for the existence of two subsets of samples that show opposite trend in the usage of exon 20 of CTNND1.

### Investigation of isoform switching: Non-subtype associated events

We identified 88 pairs of isoforms with a switching pattern that were not associated with intrinsic molecular subtype, however, we noted a biological feature that confounded this analysis. Namely for many of these apparent switching events, one of the isoforms was a very rare event that only happened in a few samples. To address this feature objectively, the splicing patterns of these 88 genes were interrogated by unsupervised k-means clustering, and only the expression of 16 isoform pairs stratified samples into two clusters in which both isoforms were detected and were inversely correlated, and where the size for both clusters was greater than 50 samples per cluster/group (Additional file 6: Table S5).

**Fig. 4:**
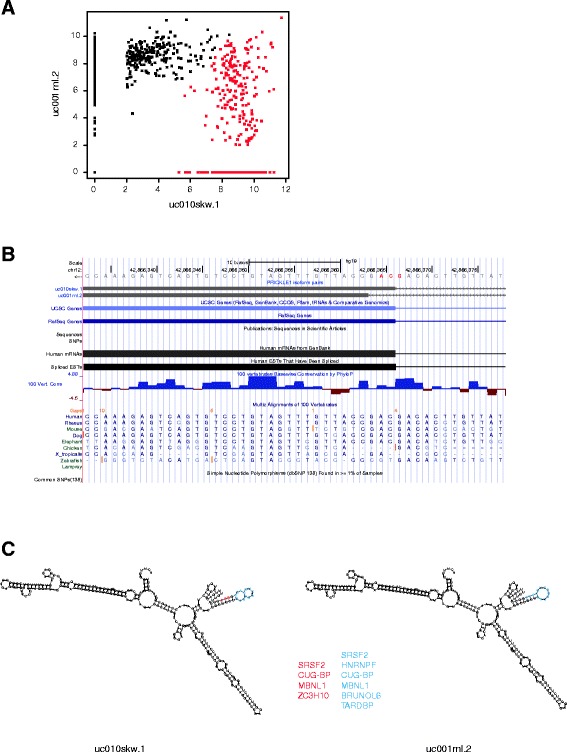
Isoform expression and gene structure of PRICKLE1. **a** Expression pattern of PRICKLE1 isoforms uc010skw.1 and uc001rnl.2. Two expression patterns (red and black) were identified by K-means clustering in subsets of breast samples. **b** PRICKLE1 gene structure.uc001skw.1 and uc001rnl.2 contain alternative 3’ splice site in exon 7 (red). **c** 5’ UTR structure of PRICKLE1 and putative RNA binding proteins. Alternative 3’ splice site (red) and hairpin structure at the flanking region (blue) are enriched with putative binding sites of RNA binding proteins

As an example, we further investigated the isoform pair uc010skw.1/uc001rnl.2 of PRICKLE1. While the gene expression level of PRICKLE1 was not significantly different between tumor and normal samples, two subpopulations were identified based on the isoform pattern (Fig. 4a). The two clusters have comparable RIN scores, excluding the possibility that the differential expression of transcripts was confounded by partial degradation. The alignment of the two transcripts was validated by *de novo* assembly of the samples with significant differential expression of PRICKLE1 isoform pair (Additional file 9: Figure S2B).

PRICKLE1 has an alternative 3’ splice site with isoform uc001skw.1 containing three additional nucleotides in exon 7 compared to isoform uc001rnl.2 (Fig. 4b). Exon 8 and part of exon 7, including the alternative splice site, constitute the 5’ UTR sequence of PRICKLE1. While both splice sites were validated by contigs from *de novo* assembly, the smaller exon 7 was only assembled in the subset of samples that are enriched with uc001rnl.2. These two transcripts have no difference in the coding regions, however, a closer scrutiny of the PRICKLE1 sequence reveals that the alternative usage of this splice site affects the structure of 5’ UTR. An internal loop is predicted to be introduced in the hairpin structure of uc001skw.1 (Fig. 4c). Moreover, several binding motifs of **R**NA-**B**inding **P**roteins (RBP) were identified in the alternative 3’ splice site and the loop region (Fig. 3c). One of the RBP potentially being affected is SRSF2, which regulates splicing switches between pro- and anti-apoptotic isoforms of at least four genes: c-flip, caspase 8, caspase-9 and BCL-X [3, 8]. Of them, the abundance of CASP8 isoform uc010ftc.1 was associated with this PRICKLE1 isoform pattern (data not shown). These results suggest that the RBP binding in the 5’ UTR region potentially contributes to the regulation of PRICKLE1 function or alters it’s interaction with other genes.

**Fig. 5:**
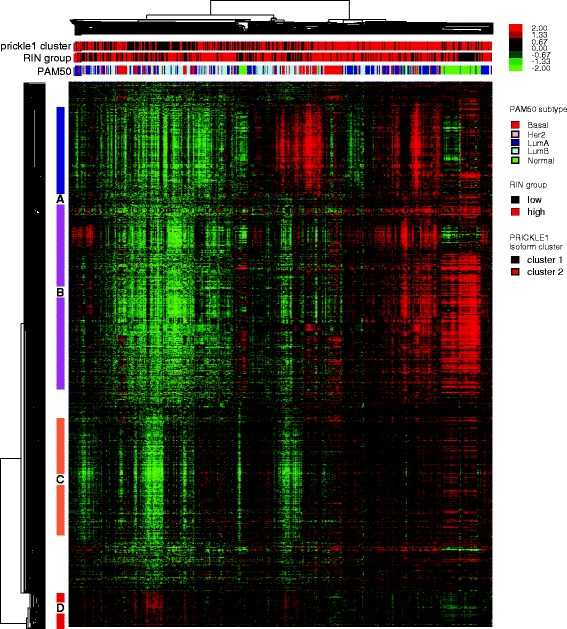
Hierarchical clustering analysis of the gene signatures associated with the isoform expression pattern of PRICKLE1. 1059 genes are significantly correlated with the ratio of uc010skw.1 and uc001rnl.2, and are not associated with PAM50 subtype or RIN score. PRICKLE1-based clusters identified by K-means clustering were color coded consistent with Fig. 3a. RIN group displays samples with high (>8.7, median RIN) and low RIN score (≤8.7). Four gene expression signatures (**a**-**d**, shown by side color bars) are identified to be involved in cancer hallmarks

We next assessed the potential effect of this PRIKLE1 alternative splicing event on global expression patterns via supervised gene expression analysis using Significance Analysis of Microarrays/SAM [26]. The expression ratio of uc001skw.1 versus uc001rnl.2 was used as the supervising parameter and was significantly correlated with 1059 genes (FDR = 0; termed PRICKLE1-alt genes) (Additional file 10: Table S8). We then used these 1059 genes in a hierarchical clustering analysis across the 819 samples and identified 4 distinct gene expression signatures (Fig. 5).

Gene cluster A, which was more highly expressed in tumors with the PRICKLE1 uc010skw.1 isoform, was enriched with genes involved in inflammatory response and activation of immune response (Fig. 5). A potential tumor suppressor gene SYK is in this cluster; SYK has been identified as a regulator of epithelial cell growth and its splicing pattern alters cell survival in breast and ovarian cancer [27]. Gene cluster B was highly enriched with genes of extracellular matrix and/or genes that regulate signal transduction pathways for Epithelial-to-Mesenchymal transition (EMT), including RBFOX2 (RBM9), which has been demonstrated to regulate the EMT splicing in a panel of breast cancer cell lines and primary tumors [28, 29]. Other intriguing genes included JAK1 that encodes a protein tyrosine kinase that mediates the cytokine receptor signaling via activation of STAT transcription factor. Interestingly, JAK2 was also selected in the PRICKLE1-alt signature, which regulates STAT-independent oncogenic pathway in addition to the JAK/STAT pathway [30]. Other cancer-associated genes in Cluster B include ZEB2, NOTCH1, and FAT1.

Gene cluster C was enriched with genes that regulate phosphorylation and transcription. Two serine/threonine protein kinases, ATM and MAP3K2, were identified, suggesting its involvement in DNA repair and apoptosis pathways. Cluster D contained genes with function in oxidative phosphorylation and ATP synthesis coupled electron transport. Prior studies have revealed that deregulated energy metabolism, such as glycolytic switch, is an emerging hallmark of cancer [31]. Therefore, genes of cluster D are potentially involved in reprograming of energy metabolism in cancer cells to support cell growth and division. None of these gene signatures, nor the expression of PRIKLE1 itself, were prognostic.

## Discussion

Changes in alternative splicing could lead to, or contribute to, tumor formation. Genomic studies have revealed that more than 15,000 isoforms are associated with several cancer types [18, 29]. In this study, we systematically analyzed all the annotated transcripts/isoforms in UCSC database in 819 TCGA breast tumor and normal samples assayed by RNA-seq. Despite the increased complexity of transcripts compared to genes, on average only 2.2 isoforms per gene were detected. However, a few genes produced highly complex splicing patterns, indicating their potential regulation on the splicing level. Past work has established links with cancer for some of these genes; for example, NCOR1, a nuclear co-repressor with 14 isoforms detected, has been postulated to regulate retinoic acid and thyroid hormone receptor protein levels and to disrupt PPARα/γ signaling in prostate cancer [32].

For most genes, including those with complex splicing patterns, the expression levels of genes and isoforms from the same gene were positively correlated across these samples, even in genes with low levels of expression. On a first pass analysis to identify genes showing isoform switching (i.e. negatively correlated isoforms), 78 % of inversely correlated isoforms showed significant difference in RNA integrity (i.e. RIN scores), although no association was observed between the isoform correlation and isoform lengths. Of note, 636/819 samples had a RIN score of at least 8, which suggests that even for high-quality samples, investigation of relative expression of transcripts must still account for RNA integrity. In our study, we adopted a computational strategy to minimize the effect of RIN. An alternative approach may be to use RNA-seq library preparation protocols that don’t rely on polyA enrichment, such as Ribo-Zero with random priming. We observed in our previous studies that Ribo-Zero provided less biased 5’-to-3’ coverage, even in FFPE samples [33].

Based on the global expression profile and after accounting for RIN, we identified 470 pairs of predominant and alternative isoforms that showed “switching” events; most of them were associated with intrinsic molecular subtypes. As exemplified by CTNND1, the switching pattern between basal-like and luminal breast tumors was similarly observed in the PanCan12 Squamous Genomic Group versus PanCan12 Lung Adenocarcinoma-enriched Group, which is indicative of another common characteristic of breast basal-like and squamous tumor subtypes [25]. CTNND1, also known as p120, has been identified to provide both oncogenic and tumor suppressor functions. p120 regulates the turnover of cadherin and maintains the stability of adherins junctions at the plasma membrane [34]. In addition, it also modulates Rho GTPase activity as an inhibitor or activator depending on the cellular context [35, 36]. p120 can also translocate to the nucleus and interacts with the transcriptional repressor Kaiso [37], relieves the repression of its target genes such as WNT11 and CyclinD1 [38, 39], and indirectly modulates the Kaiso-dependent sequestering of β-catenin and TCF/LEF transcription factor. Therefore, the localization of p120 in specific cellular compartments is likely to be functionally relevant. Four ATG start sites located at amino acids 1, 55, 102, 324 initiate the expression of isoforms 1–4 in CTNND1. Three other exons (exon 18(A), exon 20(B) and exon 11(C)) are also alternatively included in the transcripts [40]. A nuclear export sequence (NES) is located at exon 20(B). While emerging interest has been focused on the alternative N-terminal and the regulatory phosphorylation domain, the function of nuclear localization sequences (NLS) and NES in p120 is still unclear.

Here we identified a subtype-associated switching event of CTNND1 isoform 3A(uc001nlt.3) and isoform 3AB(uc001nlo.3), in which isoform 3A(uc001nlt.3) is predominantly expressed in the breast basal-like subtype and the PanCan12 Squamous group. We hypothesize that it potentially suggests the involvement of exon B/NES sequence in regulating nucleo-cytoplasmic shuttling activity. Past work has demonstrated that nuclear Kaiso expression is enriched in basal-like/triple-negative breast cancers and in BRCA1 associated invasive breast cancer, and is inversely correlated with cytosolic p120 [41]. Here, we showed that isoform 3A, which lacks the exon B/NES domain, is enriched in the basal-like subtype. Collectively, it suggests the link between the missing of exon B and the low cytosolic p120 level. Of course, additional experiments are required to validate the role of NES, but our study here reveals that, in addition to the balance of isoform 1 and 3 that is known to be critical for cell motility, the ratio of an isoform with or without NES might also affects the localization and consequently the function of p120.

In contrast to switching events correlating with subtype, many fewer were seen that were independent of subtype (i.e. 88 pairs). The switching of PRICKLE1 uc010skw.1 and uc001rnl.2 was one such event. Interestingly, PRICKLE1 isoform supervised gene expression analysis revealed its correlation with >1000 genes (Fig. 5) including tumor suppressors, transcription regulators, and genes that are crucial for EMT and energy metabolism. Previous studies demonstrated that PRICKLE1 regulates two pathways in cancer. In hepatocellular carcinoma (HCC), PRICKLE1 negatively regulates Wnt/β-Catenin pathway by binding to DVL3 and facilitating its ubiquitination/degradation [42]. Alternatively, in chronic lymphocytic leukemia (CLL), PRICKLE1 mediates migration and transendothelial invasion of CLL cells via DVL3-independent Wnt/planar cell polarity (PCP) signaling pathway [43]. None of the PRICKLE1-alt gene clusters we identified showed an enrichment of genes of the Wnt/β-Catenin or Wnt/PCP pathway. Moreover, the alternative splice site involved in the switching does not disrupt the protein coding sequences. Computational analysis of the transcript sequences provided a lead for its post-transcriptional regulation; namely the alternative splice site at the 5' UTR region, and the hairpin structure of the flanking region, are enriched with putative RBP binding domains, and thus in breast cancer PRICKLE1 function may be regulated by alternative RBP binding abilities due to alternative stem-loop structures in the 5’ UTR.

## Conclusions

In summary, we provide a global profile of annotated transcripts in breast tumors and normal samples, and our data demonstrate the general agreement in isoform and gene level expression. Isoform switching events were identified, but most correlated with subtype, and only a minority were subtype-independent. These results identify many novel genomic events that are involved in breast tumor biology and detail for the first time, the precise alternative splicing events that occur in breast cancer and that eluded the field before the advent of RNA-sequencing.

## Methods

### Read processing and alignment

RNA-sequencing data for all TCGA BRCA and TCGA PanCan samples were obtained from https://tcga-data.nci.nih.gov/tcga/. All samples were sequenced using previously published methods [25]. The alignment and quantification was performed by MapSplice [44] v12_07 and RSEM [21] v1.1.13 respectively, using the reference transcriptome of UCSC hg19 GAF2.1 [45] for known genes, as described in [33]. Data was normalized to a fixed upper quartile within each sample.

### Quantification of isoform abundance

The transcript/isoform abundance was filtered by requiring the RSEM normalized count to be ≥3 for each transcript. The detected transcript sets were defined as transcripts that were reported present in >60 % samples and with 3 or more normalized reads. The log2 transformed transcript abundance was reported. For each gene, the predominant isoform was determined as the transcript that provided the greatest upper quartile value across all samples.

### Identification of isoform switching events

The isoform switching events in all samples (both tumor and normals) were identified using a linear regression model: y_gi_ ~ β_1_*x_b_ + β_2_*x_gi_, where y_gi_ is the alternative isoform, x_gi_ is the predominant isoform, x_b_ is RIN score. The events were defined as isoform pairs with (i) β_2_ < 0; (ii) *p*-value for x_b_ > 0.2 and (iii) *p*-value for x_gi_ < 0.05.

### Isoform switching events associated with subtype

The expression pattern of isoform pairs was characterized by the ratio of alternative isoform to predominant isoform. The Student’s t-test was used to estimate the correlation of the isoform pairs and tumor subtype as previously determined by the PAM50 algorithm [24, 25], with the significance determined at *p*-value < 0.05.

### Validation and analysis of the sequence of isoform switching pairs

For each isoform of the switching pairs of CTNND1 and PRICKLE1, 10 samples with the greatest relative abundance were selected. Reads mapped to the gene of interest were pooled and *de novo* assembled using Trinity [46]. Assembled contigs were then mapped to the genome by BLAT [47] and visualized by IGV [48]. The annotation of transcript sequence and the structure of 5’ UTR sequence of PRICKLE1 were retrieved from UCSC genome browser [45]. RBPmap [49] was then used to predict the RBP binding sites in the PRICKLE1 5’ UTR region.

### Differential gene expression associated with isoform switching pairs

The expression pattern of PRICKLE1 transcript of uc001rnl.2 and uc010skw.1 was profiled, and two subpopulations of samples with differential usage of PRICKLE1 isoforms were identified by K-means clustering (Fig. 4a). Quantitative SAM analysis was performed to identify genes that showed differential expression associated with the ratio of PRICKLE1 transcript of uc001rnl.2 to uc010skw.1. The gene list was obtained with a FDR of 0 (Additional file 10: Table S8). Four gene signatures were identified by the hierarchical clustering analysis and its functional annotation was performed by DAVID Tools [22].

## Ethics statement

This study used de-identified human data from a public data portal, and is thus not considered to be human subjects research. Ethics approval is not required for the study of data that is not human subjects research.

## Additional files

Additional file 1: Table S1. Patient characteristics of 728 breast tumors and 91 normal samples of TCGA BRCA dataset. (XLSX 54 kb) Additional file 2: Table S2. 43 genes with more than 10 expressed and detected transcripts per gene. (XLSX 10 kb) Additional file 3: Table S3. Functional annotation clustering of 43 genes with complex splicing patterns. Functional annotation clusters are sorted by the overall enrichment score based on the EASE score, a modified Fisher Exact *P*-value, of annotation terms. Genes associated with each term and the EASE Score are reported. Comparison of annotation terms associated with the top six clusters is coded as the following: Genes associated with specific terms are coded as 1; genes with no involvement are coded as 0. (XLSX 22 kb) Additional file 4: Figure S1. Identification of isoform switching events and significance of RIN score. (A) Hierarchical clustering of 2110 pairs of predominant/alternative isoforms that are inversely correlated. Two groups of isoforms showed significantly high and low expression in the cluster of samples with low RINs. (B) Two clusters of breast samples identified by the 2110 isoform pairs display significant difference in the RIN score. (C) Based on the linear regression model, the estimated coefficients of two variables, the RIN score and the predominant isoform, of all isoform pairs (light gray), 2110 inversely correlated pairs (dark gray) and 470 switching pairs (black). (PDF 750 kb) Additional file 5: Table S4. List of 917 isoform switching pairs associated with significant up and down regulation in sample with low RIN scores. (XLSX 86 kb) Additional file 6: Table S5. 470 pairs of isoforms with switching events in TCGA BRCA dataset. For each isoform pair, two clusters of samples with differential isoform expression pattern was identified by K-means clustering; and was summarized on the complete data set (K-means cluster sample size (overall)) and on the subset in which both isoforms were detected (K-means cluster sample size (detected)). (XLSX 63 kb) Additional file 7: Table S6. Functional annotation clustering of 452 genes involved in isoform switching events. Functional annotations clusters are sorted by the overall enrichment score based on the EASE score. Genes associated with annotation terms, EASE score and comparison of terms in the top six clusters are presented. (XLSX 89 kb) Additional file 8: Table S7. 229 pairs of isoforms switching events associated with luminal vs. basal subtype. The correlation of isoform expression pattern with luminal vs. basal subtype was identified in the TCGA BRCA dataset, and was tested in comparing two sets of tumor cluster pairs in TCGA PanCan dataset: (a) LUAD and Squamous, (b) Luminal BRCA and Basal BRCA. (XLSX 49 kb) Additional file 9: Figure S2. Validation of the sequence of (A) CTNND1 and (B) PRICKLE1 transcripts by *de novo* assembly. (PDF 578 kb) Additional file 10: Table S8. Significantly differentially expressed genes associated with the ratio of PRICKLE1 uc010skw.1 and uc001rnl.2. 1059 genes were identified by qualitative SAM analysis using a FDR of 0. And four gene signatures are enriched with cancer genes. (XLSX 89 kb)

## Abbreviations

FDR: false discovery rate
SAM: significant analysis of microarray
