# Supplementary material for: Identification of mRNA isoform switching in breast cancer
Source: BMC Genomics. 2016 Mar 3;17:181. doi: 10.1186/s12864-016-2521-9 (PMC4778320; doi:10.1186/s12864-016-2521-9)

Supplementary figure 1. Identification of isoform switching events and the significance of RIN score.

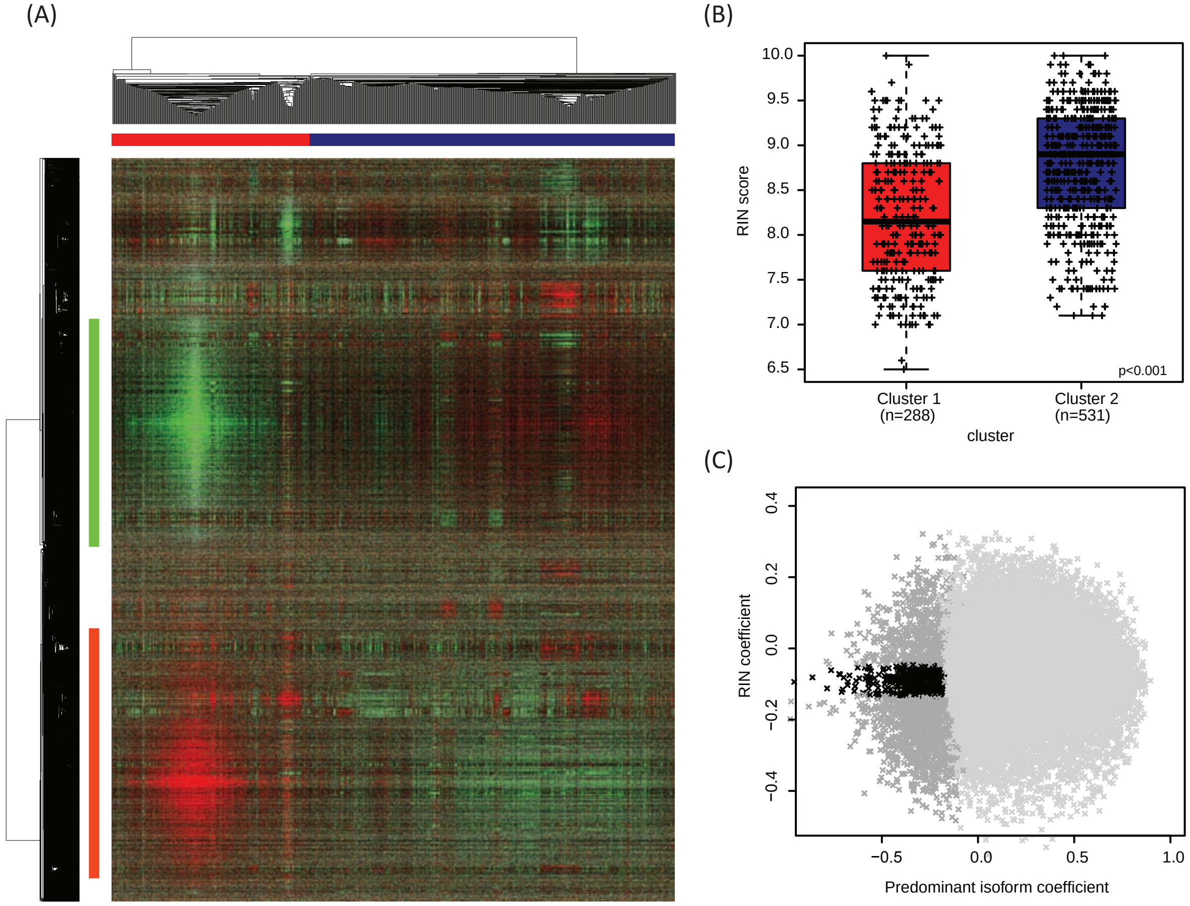

Supplement: Additional file 4: Figure S1. — Identification of isoform switching events and significance of RIN score. (A) Hierarchical clustering of 2110 pairs of predominant/alternative isoforms that are inversely correlated. Two groups of isoforms showed significantly high and low expression in the cluster of samples with low RINs. (B) Two clusters of breast samples identified by the 2110 isoform pairs display significant difference in the RIN score. (C) Based on the linear regression model, the estimated coefficients of two variables, the RIN score and the predominant isoform, of all isoform pairs (light gray), 2110 inversely correlated pairs (dark gray) and 470 switching pairs (black). (PDF 750 kb) [file 12864_2016_2521_MOESM4_ESM.pdf]
