# Supplementary material for: Identification of mRNA isoform switching in breast cancer
Source: BMC Genomics. 2016 Mar 3;17:181. doi: 10.1186/s12864-016-2521-9 (PMC4778320; doi:10.1186/s12864-016-2521-9)

Supplementary figure 2. Validation of the sequence of CTNND1 and PRICKLE1 transcripts by *de novo* assembly.

(A) CTNND1 isoform pairs of uc001nlt.3 and uc001nlo.3.

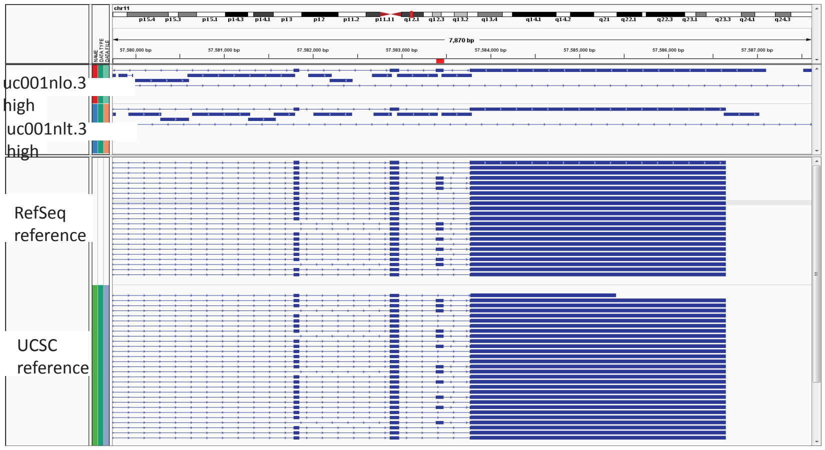

(B) PRICKLE1 isoform pairs of uc010skw.1 and uc001rnl.2

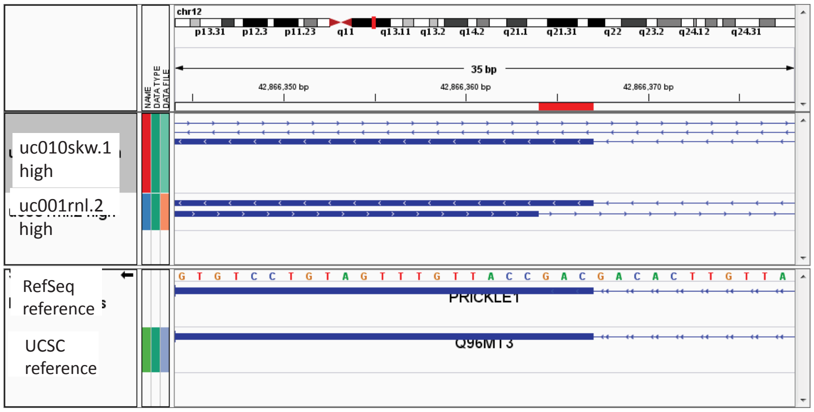

Supplement: Additional file 9: Figure S2. — Validation of the sequence of (A) CTNND1 and (B) PRICKLE1 transcripts by de novo assembly. (PDF 578 kb) [file 12864_2016_2521_MOESM9_ESM.pdf]
